# Submicron Nonporous Silica Particles for Enhanced Separation Performance in pCEC

**DOI:** 10.3390/molecules28083542

**Published:** 2023-04-17

**Authors:** Qing Liu, Chao Yan, Yan Wang

**Affiliations:** 1 Hubei Province Key Laboratory of Occupational Hazard Identification and Control, School of Medicine, Wuhan University of Science and Technology, Wuhan 430065, China; lqing@wust.edu.cn; 2 School of Pharmacy, Shanghai Jiao Tong University, Shanghai 200240, China

**Keywords:** submicron scale particles, pressurized capillary electrochromatography, separation

## Abstract

Applications of submicron-scale particles are of rising interest in separation science due to their favorable surface-to-volume ratio and their fabrication of highly ordered structures. The uniformly dense packing beds in columns assembled from nanoparticles combined with an electroosmotic flow-driven system has great potential in a highly efficient separation system. Here, we packed capillary columns using a gravity method with synthesized nanoscale C18-SiO_2_ particles having diameters of 300–900 nm. The separation of small molecules and proteins was evaluated in the packed columns on a pressurized capillary electrochromatography platform. The run-to-run reproducibility regarding retention time and peak area for the PAHs using a column packed with 300 nm C18-SiO_2_ particles were less than 1.61% and 3.17%, respectively. Our study exhibited a systematic separation analysis of small molecules and proteins based on the columns packed with submicron particles combined with the pressurized capillary electrochromatography (pCEC) platform. This study may provide a promising analytical approach with extraordinary column efficiency, resolution, and speed for the separation of complex samples.

## 1. Introduction

Nanotechnology is rapidly growing in many scientific fields, including chemistry, material sciences, physics, medicine, and electronics [1,2]. Materials in the range of nanometers to micrometers have been extensively studied in separation science in recent years [3,4,5,6]. The large surface area and highly ordered structure of small particles make them potent for use as chromatographic materials achieving highly efficient separation, according to the van Deemter equation [7]. Moreover, slip flow was proven to occur in a nanoscale channel made of submicron silica spheres in reversed-phase liquid chromatography, providing an additional improvement in separation efficiency by increasing volume flow rates and reducing the velocity distribution in the mobile phase [8,9,10].

In published studies, minimal plate heights were obtained using nonporous silica particles in reversed-phase liquid chromatography (RPLC). B. Wei [8] reported a plate height of only 15 nm for BSA on a column with a length of 12 mm packed with 470 nm particles inside of 75 μm i.d. fused silica capillaries at room temperature. Malkin, D.S. [11] reported that DiI-C(12) exhibited plate heights as low as 0.23 μm using 75 micron i.d. capillaries packed with 330 nm silica particles. Liao [12] packed 2.5 mm photonic crystals into microfluidic channels, which separated amino acids in 4 s. Wei [13] packed a silica colloidal crystal column with 330 nm nonporous silica spheres inside of a 75 μm i.d. capillary, and a plate height of less than 50 nm was obtained for the lysozyme. Increasingly more microfluidic techniques based on micro/nanoscale materials have been developed in the field of separation science, though most of the studies have focused only on biomacromolecules, such as proteins and nucleic acid. Fewer studies have systematically shown the separation of both small molecules and proteins using a column filled with various-sized nano silica particles.

Although the separation efficiency of a column packed with submicron particles is relatively high, the columns are extremely short in length (typically shorter than 20 mm), and they are typically applied in chip channels or coupled with mass spectrometry [12,13,14,15,16], which has been shown to limit the application of a column packed with submicron particles. Thus, a packing column with a longer length is urgently needed. However, a longer column packed with submicron particles will introduce the main negative effect in LC: back-pressure [17]. In addition, most commercial instruments have not been designed for use with a column packed with submicron particles.

pCEC is a separation technique which combines the high efficiency of capillary electrophoresis (CE) with the selectivity offered by LC [18,19,20]. pCEC provides high separation, high selectivity, high resolution, and fast speed for separation. The mobile phase is mainly driven by the electroosmotic flow (EOF), which is created from the electrical double layer and acts as a pump in the capillary column. The pressure pump is designed to prevent bubble formation in the column and to program the composition of the mobile phase. Combining nonporous submicron columns with a pCEC platform could overcome the issue of high back-pressure in the system and avoid the column dry-out problem caused by a CE system. This may provide a promising analytical approach with extraordinary column efficiency, resolution, and speed for separation science.

In this work, monodispersed homogeneous nanoscale silica particles with diameters of 300, 420, 500, 620, and 820 nm were synthesized and derivatized with n-octadecyltrichlorosilane (ODS) and methyl trichlorosilane, and then they were packed into 100 µm i.d. capillary columns. pCEC, coupled with a UV/Vis detector, was applied to evaluate the separation efficiency of both the small and large molecules on these packed columns. An analysis of the aromatic compounds, PAHs, estrogens, and proteins (lysozymes, cytochrome C, ribonuclease A, and ovarian cancer anti-idiotypic mini bodies) was conducted on the system in which the pCEC was coupled with a column packed with submicron particles. The separation efficiency of a column packed with particles of different sizes was evaluated and compared. Column efficiencies of 273,000 plates (plate height, H = 0.37 μm) for hexoestrolum (HE) and of more than 100,000 plates per column (plate height of < 1 μm) for protein were achieved. Our work introduced the systematic preparation and application of a column packed with synthesized submicron particles. The separations conducted on columns packed with 300–900 nm particles were evaluated and compared. This work enhances the application of submicron packing materials, providing an opportunity for the further development of high-efficiency technologies in separation.

## 2. Results and Discussion

### 2.1. Characterization

**Figure 1:**
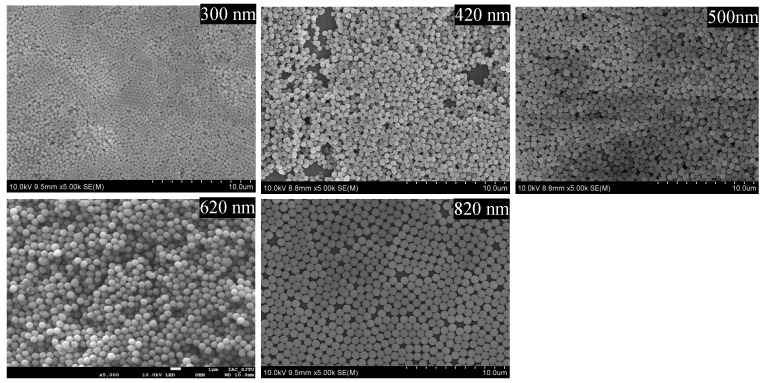
SEM images of synthesized SiO_2_ particles with diameters of 300 nm, 420 nm, 500 nm, 620 nm, and 820 nm.

**Figure 2:**
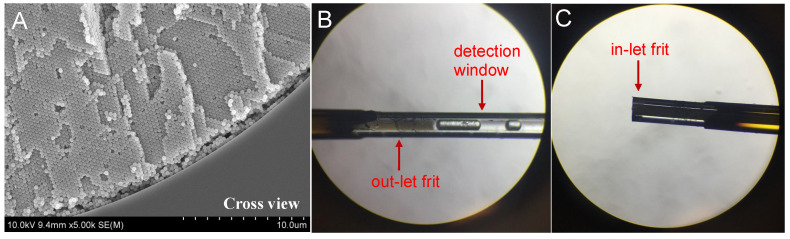
(**A**) SEM images of the cross-view of a 100 μm i.d. capillary packed with 420 nm particles. (**B**,**C**) Photo of a column packed with submicron particles under a microscope: (**B**) outlet frit and detection window and (**C**) inlet frit tip.

The uniformity of the particles and packed column is critical for high-efficiency separation, and it was also the most challenging element of our project. A scanning electron microscope (SEM) test was applied to confirm the uniformity of the submicron SiO_2_ particles and the packed column. The SEM image in Figure 1 shows the overall morphologies of the synthesized particles with 300–900 diameters, and they were primarily monodispersed particles. Figure 2A shows an SEM image of a cross-section of the capillary where the face-centered cubic domains were observed. Figure 2B,C show side views of the packed capillaries with submicron particles under a microscope, which revealed the expected uniform packing of the submicron particles.

### 2.2. Optimization of the Preparational Method for a Packed Column

To obtain C18-modified SiO_2_ particles, the synthesized sphere was modified with octadecyltrichlorosilane and methyl trichlorosilane [6,14]. The column was packed by filling the particle suspension into the capillary, and in our study, the column length could be flexibly adjusted by changing the concentration of the sub-microsphere suspension.

**Figure 3:**
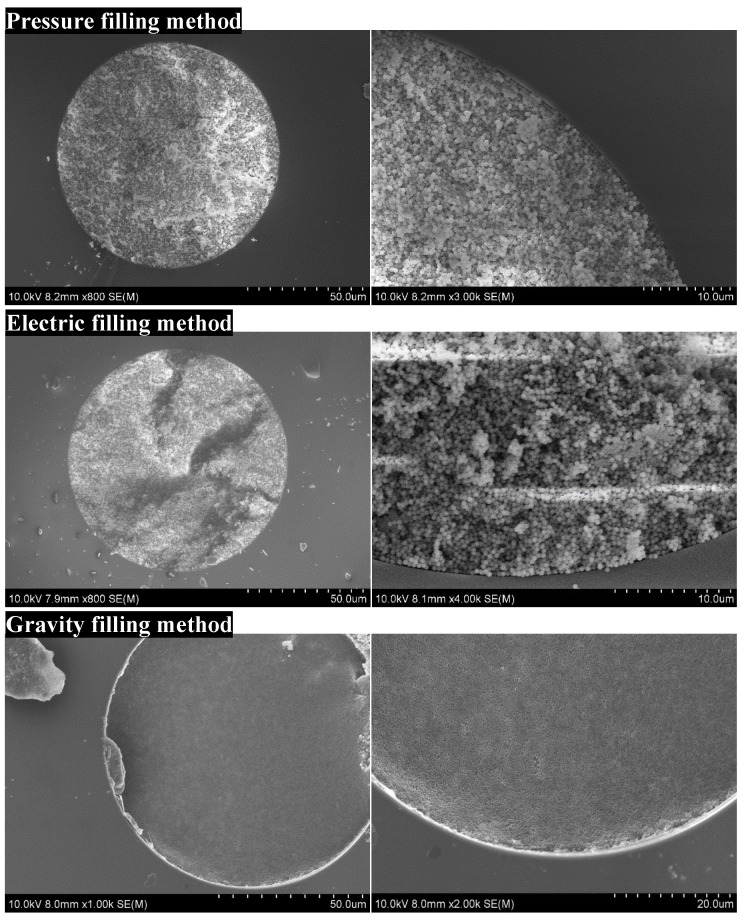
Cross sections of the capillary columns obtained by the pressure filling, electric filling, and gravity sedimentation self-assembly methods, respectively.

The approach used to fill the capillary with particles is the key to obtaining a column packed with sub-microspheres. We investigated several packing methods, including the pressure filling, electric filling, and gravity filling methods (Figure 3). Pressure filling is typically conducted for the packing of stainless-steel columns and capillary columns [21]. A temporary inlet frit was made at the end of the capillary by packing same-sized silica particles into a capillary with a 1 mm length, followed by sintering with a thermal stripper (Unimicro Technologies, Pleasanton, CA, USA). A pressure pump set at 6000 psi was employed to fill the column with a particle suspension. Specifically, the homogenizer, which was connected with the pump, was filled with the particle suspension, and then the particle suspension was sent into the capillary under pulsed pressure, where the solvent flowed out of the plunger under pressure. The SEM image in Figure 3 shows the disordered stacking structure in the column packed by the pressure filling method, where the large gaps between microspheres introduced peak broadening during separation. This observation suggested that the pressure filling method was not a good choice for packing the submicron particles. For electric filling, we employed an electrophoresis device to fill the capillary with the particle suspension. The particles were charged in a water/ethanol solution and moved into the capillary in a certain direction. The assembled bed was supposed to be tightened and uniform with the existing EOF [22,23]. However, the particles were disordered in the column when we packed it using this method. This may be attributed to the submicron size of the particles, which made it difficult to arrange them in a tight and orderly manner. For the gravity filling method, the settlement of the particles was driven by the force of gravity [24,25,26]. This procedure can take 3–7 days, and it took much longer than the other methods mentioned above. As a result, the particles were highly ordered and packed uniformly inside the capillary using the gravity settling method, according to the SEM image shown in Figure 3.

Following the gravity settling of the submicron particles, the column was pressurized at 10,000 psi pressure by a pump, the frit was sintered, and the detection window was created with a thermal stripper, as shown in Figure 2B,C. The frit and detection window were made strictly flat to ensure the high efficiency of the column.

In summary, the gravity filling method is the most economical and simple among these methods for fabricating submicron particles, and no special equipment is required. The slow settlement was driven by gravity, allowing the sub-microspheres to completely self-assemble and pack densely in the capillary.

### 2.3. Separation of the Aromatic Compounds

**Figure 4:**
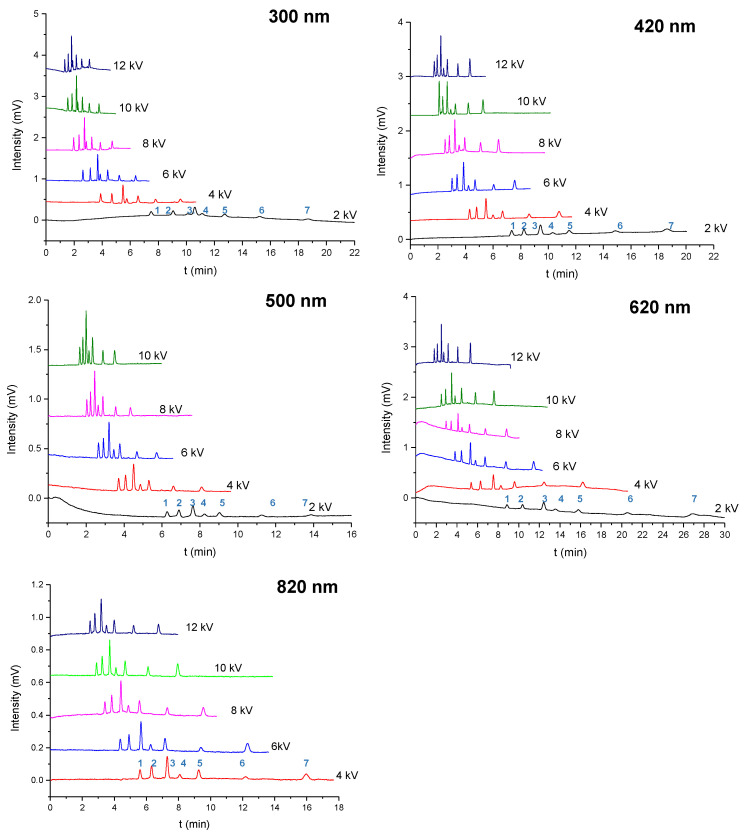
The pCEC separation of the thiourea and 6 aromatic compounds at voltages of 2 kV to 12 kV in the columns packed with 300, 420, 500, 620, and 820 nm particles. The experimental conditions were as follows: column: 100 μm i.d. × 100/300 mm (effective/total length); pressure = 15.5 MPa; mobile phase: acetonitrile—10 mM phosphate butter (70:30, *v*/*v*); pH = 7.8; and split ratios of 800:1. The peaks correspond to the following: (1) thiourea; (2) a-naphthol; (3) benzophenone; (4) naphthalene; (5) biphenyl; (6) butylbenzene; and (7) phenylcyclohexane.

The separation based on the column packed with submicron particles was supposed to exhibit a higher column efficiency than a commercialized column. Figure 4 shows the separation of the 6 aromatic compounds and thiourea at voltages of 2 kV to the upper limitation (10 kV or 12 kV) using pCEC with columns packed with particles that were 300 nm, 420 nm, 500 nm, 620 nm, and 820 nm in diameter. The highest voltage for the column packed with 500 nm particles was 10 kV since the current did not go up when we set it to 12 kV, which may be attributed to an instrument or column limitation. The baseline separation of the six compounds was achieved with a good peak shape at a voltage of >4 kV. The separation in the column packed with smaller particles and at a higher voltage resulted in a better peak shape and higher efficiency. Seven compounds were separated in 3.5 min at a voltage of 12 kV in a column packed with 300 nm particles, which took only one-fifth as long as the separation at voltage of 2 kV in a column packed with 300 nm particles and only half as long as the separation at a voltage of 12 kV in a column packed with 820 nm particles. Consistently, our previous study revealed that the EOF velocity in a column packed with 300 nm particles is twice the EOF velocity in a column packed with 820 nm particles, suggesting that the EOF primarily served as a driving force for the mobile phase compared to the pressure in the CEC with submicron particles.

As shown in Figure S1, the column efficiency was compared across the columns packed with the submicron particles. The separation efficiency reached 218,000 plates/m (6.387 min) for phenylcyclohexane using the column packed with 300 nm particles at a voltage of 6 kV, an efficiency of 157,000 plates/m (5.088 min) was achieved for the butylbenzene probe at a voltage of 8 kV when the column was packed with 420 nm particles, the column packed with 500 nm particles could generate 152,000 plates/m (8.088 min) for the separation of phenylcyclohexane using a voltage of 6 kV, an efficiency of 239,000 plates/m (5.303 min) was obtained for phenylcyclohexane at a voltage of 12 kV in a column packed with 620 nm particles, and an efficiency of 197,000 plates/m (6.76 min) was achieved for phenylcyclohexane at a voltage of 8 kV when the column was packed with 820 nm particles. The column efficiency reached as high as 200,000 plates/m when the column was packed with 300 nm, 620 nm, and 820 nm particles, among which the column packed with 300 nm particles generated the best efficiency for the separation of aromatic compounds, indicating that column efficiency is negatively correlated with particle size, even at the micron scale. For comparison [27], the retention time of naphthalene on a pCEC platform using a column packed with 1 mm C18-bonded silica particles was 25.01 min, which was more than 13 times longer than the retention time observed (1.818 min) in our study [27]. These data indicate that using smaller-sized sub-microspheres for packing benefits the separation efficiency on a pCEC platform.

### 2.4. Separation of PAHs

Polycyclic aromatic hydrocarbons (PAHs) are produced by the incomplete combustion of petroleum, automotive emissions, and smoking. PAHs are commonly detected in food and the environment, exhibiting a great risk to human health. In our study, the separation of eight PAHs was conducted using the prepared column. Our previous work observed the separation of 8 PAHs (naphthalene, acenaphthylene, fluorene, phenanthrene, anthracene, fluoranthene, benzanthracene, and benzofluoranthene) in a column packed with C18-bonded 420 nm particles [6], and here, we achieved the separation of 8 PAHs in columns packed with C18-bonded 300, 500, 620, and 820 nm particles. The electrochromatograms are shown in Figure S2, the column efficiency is shown in Figure S3, and the separation repeatability of the eight compounds using five columns is shown in Table S1. Eight compounds could achieve baseline separation in 7 min with good peak shapes and repeatability with an applied voltage on columns packed with 300, 500, 420, and 620 nm particles. The separation in the column packed with 820 nm particles took longer (9 min), which may be attributed to the lower EOF velocity of the column. Notably, the column efficiency reached 246,000 plates/m for fluoranthene at a voltage of 8 kV when the column was packed with 300 nm particles, and 255,000 plates/m was achieved by fluoranthene at a voltage of 10 kV using a column packed with 620 nm particles. The RSDs (%) of the PAHs for the 3 runs were less than 1.61 (3.17), 0.75 (2.29), 0.05 (4.05), 0.40 (7.40), and 1.30 (6.09) for the retention time (peak area) for the columns packed with particles 300, 420, 500, 620, and 820 nm in diameter, respectively (Table S1), which demonstrated good repeatability. The column-to-column repeatability was less than 5.52% [6], which was determined in our previous study by the column packed with 420 nm particles.

### 2.5. Separation of Estrogens

**Figure 5:**
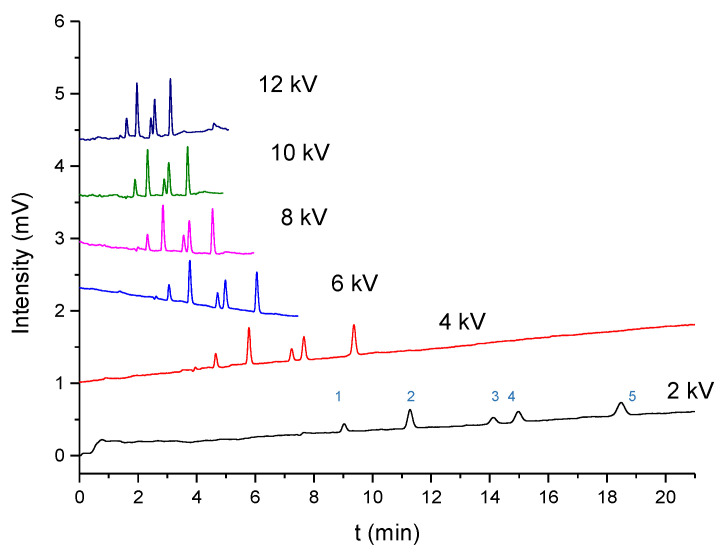
The pCEC separation of 5 estrogens at voltages of 2 kV to 12 kV in the columns packed with 300 nm particles. The experimental conditions were as follows: column: 100 μm i.d. × 100/300 mm (effective/total length); pressure = 15.5 MPa; mobile phase: acetonitrile—10 mM phosphate butter (60:30, *v*/*v*); pH = 7.8; and split ratios of 800:1. The peaks correspond to the following: (1) E3; (2) BPA; (3) E2; (4) E1; and (5) HE.

To further investigate the separation capacity of our packed column, 5 estrogens (estriol (E3), bisphenol A (BPA), 17β-estradiol (E2), estrone (E1), and hexoestrolum (HE)) were analyzed using a column packed with 300 nm C18-bonded particles (Figure 5). Five estrogens could be separated at baseline in 3.3 min at 12 kV, and the highest column efficiency, 273,000 plates/m (9.376 min), was obtained for HE at a voltage of 4 kV (Figure S4). The LODs (determined at an S/N ratio of 3) of the 5 compounds were 0.01 mg/L, 0.004 mg/L, 0.01 mg/L, 0.007 mg/L, and 0.004 mg/L for E3, BPA, E2, E1, and HE, respectively, which is very suitable for trace detection in food and environmental statistics.

### 2.6. Analysis of Proteins

**Figure 6:**
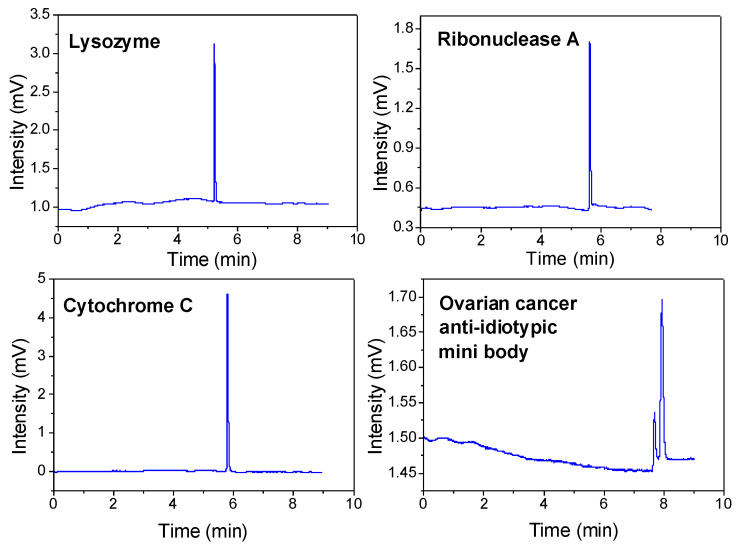
The pCEC separation of 4 proteins with 300 nm C18-bonded silica particles. The experimental conditions were as follows: column: 100 μm i.d. × 100/300 mm (effective/total length); mobile phase: acetonitrile–water (50:50, *v*/*v*); 0.1% FA; pH = 2.3; applied voltage = 10 kV (lysozyme, ribonuclease A, and cytochrome C)/8 kV (ovarian cancer anti-idiotypic mini body); pressure = 12.5 MPa; and split ratios of 1000:1. The theoretical plates results were as follows: lysozyme, 139,152; ribonuclease A, 98,973; cytochrome C, 101,156; impurity in ovarian cancer anti-idiotypic, 65,100; and ovarian cancer anti-idiotypic, 45,100.

Slip flow was found in the channel of a column packed with submicron silica spheres that were modified to be hydrophobic. Slip flow benefits protein separation efficiency by providing enhanced volume flow rates and a narrower distribution of fluid velocities. Proteins have a 10-fold slower diffusion coefficient than small molecules [8,9,10], and their optimal flow rate and plate height are much lower, providing a potentially significant advantage when using submicron particles coupled with pCEC. Figure 6 shows the electrochromatogram of the detection of lysozyme, ribonuclease A, and cytochrome C in the pCEC, where the column efficiency reached 139,152 plates (plate height = 0.72 μm), 98,973 plates (plate height = 1.01 μm), and 101,156 plates (plate height = 0.99 μm) per column, respectively, which was nearly 5 to 6 times the column efficiency observed for small molecules. This was the first time that proteins were detected using a column packed with submicron particles on a commercial pCEC-UV/Vis instrument, and the column efficiency was a milestone that had never been reached on a commercialized instrument without any modification [8,9,10], which shows enormous application potential for the separation of large biological samples.

**Figure 7:**
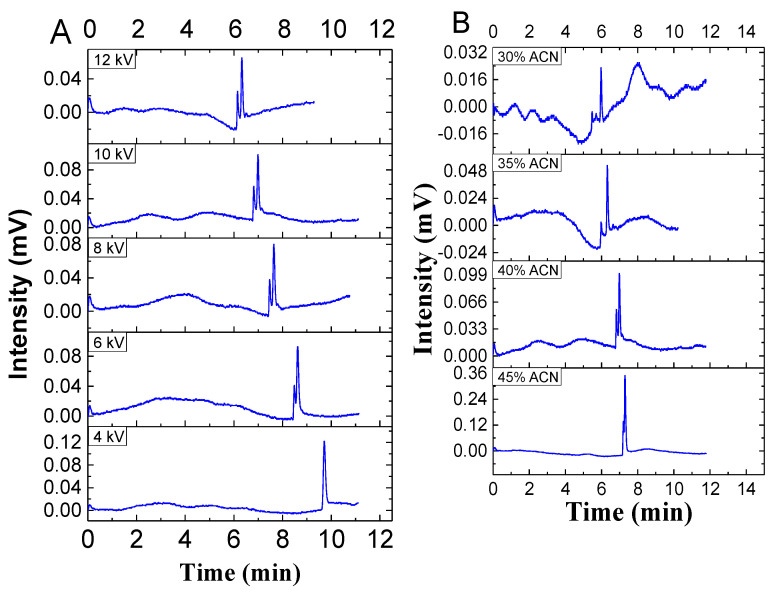
The pCEC separation of ovarian cancer anti-idiotypic mini body and impurity on 300 nm C18-bonded silica particles. The experimental conditions were as follows: (**A**) column: 100 μm i.d. × 100/300 mm (effective/total length); mobile phase: acetonitrile–water (40:60, *v*/*v*); 0.1% FA; pH = 2.3; applied voltage = 10, 8, 6, and 4 kV; and pressure = 12.5 MPa; (**B**) column: same as (**A**); mobile phase: acetonitrile–water (30, 35, 40, and 45:60, *v*/*v*); 0.1% FA; pH = 2.3; applied voltage = 10 kV; and pressure = 10.5–12.5 MPa.

Protein aggregation is very problematic as it can reduce the efficacy of protein drugs or even induce an immunogenic response in a patient. In drug development, separation plays a major role in protein drugs, as it does in small molecule drugs. In addition, ovarian cancer anti-idiotypic mini bodies were separated using this system. The two sharp peaks were well-separated with column efficiencies of 65,100 (plate height = 1.53 μm) and 45,100 (plate height = 2.22 μm) plates per column, respectively, at a voltage of 8 kV. The lower peak near the baseline was likely due to the known aggregation of this protein, and the optimization conditions are shown in Figure 7. The quite good column efficiency indicates that this type of nonporous submicron particle-packed column can be effectively used for the high-performance separation of proteins.

## 3. Materials and Methods

### 3.1. Instruments

A pCEC system (TriSep^TM^-2010, Unimicro Technologies, Pleasanton, CA, USA) coupled with an automatic sampler and an on-column UV detector were used in all the experiments. The data acquisition and analysis were conducted using TriSepTM-2003 software. The flow rate of the pumps was set at 50 μL/min, the injection volume was 1 μL, and the split ratio was set at approximately 5600:1 without the applied voltage.

### 3.2. Reagents

Liquid chromatography-grade acetonitrile (ACN) and HPLC-grade methanol (MeOH) were purchased from CNW Technologies GmbH (Germany). Analytical grade tetraethylorthosilicate (TEOS, 98%), ethanol, ammonium hydroxide (NH4OH, 28%), thiourea, organic compounds (a-naphthol, benzophenone, naphthalene, biphenyl, and butylbenzene), PAHs (acenaphthylene, fluorene, phenanthrene, anthracene, fluoranthene, benzanthracene, and benzofluoranthene), estrogens (estrone (E1), 17β-estradiol (E2), estriol (E3), 17α-ethynylestradiol (EE2), hexoestrolum (HE), bisphenol A (BPA), octadecyltrichlorosilane, and methyltrichlorosilane were purchased from Aladdin Chemistry (Shanghai, China). Analytical-grade toluene, disodium hydrogen phosphate (Na2HPO4), and sodium dihydrogenphosphate (NaH2PO4) were purchased from Sinopharm Chemical Reagent Company (Shanghai, China). Lysozyme, cytochrome C, and ribonuclease A were purchased from Aladdin Chemistry (Shanghai, China). The protein, ovarian cancer anti-idiotypic mini body, was purchased from Newsummit Biopharma Co. Ltd. (Shanghai, China). The water used was doubly distilled and purified using a Milli-Q system (Millipore, Milford, MA, USA). The aromatic compounds, PAHs, and estrogens were diluted using a mobile phase (water: acetonitrile = 3:7) to provide a solution concentration of 10^−5^ M. The protein samples were diluted using a PBS buffer (pH = 7.40) to provide a concentration of 10^−4^ M.

### 3.3. Materials

Fused silica capillaries with 100 μm i.d. and 375 μm o.d. were obtained from Yongnian Rui-feng Fiber Plant (Handan, China). Fused silica capillaries with 100 μm i.d. were pretreated by pumping 0.1 M HCl, NaOH, and ethanol for 1 h, respectively, followed by pumping ethanol for 30 min and drying under nitrogen for 1 h. Then, the capillary was used for column packing. The size and uniformity of the particles were measured using a Hitachi S-4800 (Hitachi, Japan) scanning electron microscope (SEM) equipped with a field emission gun.

### 3.4. Synthesis of the Monodispersed C18-SiO_2_ Spheres

As previously described, the monodispersed nonporous SiO_2_ spheres with diameters of between 300 and 600 nm were prepared using a slightly modified Stöber process in which TEOS was rapidly added into a mixture comprising ethanol, H_2_O, and ammonium. The monodispersed SiO_2_ spheres with diameters of 600–900 nm were synthesized using a two-phase sol–gel method. The mixture of H_2_O, ethyl alcohol, and ammonium hydroxide and the TEOS were slowly added to the reaction system separately. The particle sizes were adjusted by the ratio of each reagent. SEM images of the synthesized spherical silica particles with diameters of between 300 and 900 nm are shown in Figure 1.

The C18 modification method was conducted as described in our previous study [6]. Following calcining and rehydroxylation, the synthesized SiO_2_ particles were derivatized to the C18 and C1 phases using 10% n-octadecyltrichlorosilane (Gelest, Morrisville, PA, USA) and 10% methyl trichlorosilane in dry toluene, respectively.

### 3.5. Preparation of the Capillaries Packed with Submicron Silica Particles

The synthesized particles (300 nm, 420 nm, 500 nm, 620 nm, and 820 nm in diameter) were used to pack the columns. The preparation of the column was as described in our previous study [6]. Briefly, the capillaries were filled with the prepared particle suspension in anhydrous toluene. Temporary inlet frits were made at the ends of the capillaries, and then, the silica particles were assembled inside the capillaries using the gravity settling method. Highly ordered particle structures were formed inside the capillaries using the gravity settling method. The packed beds were tamped by pumping ultrapure water under 10,000 psi with a frit. New inlet frits were made at the ends of the capillaries at 9000 psi, and then, the temporary frits were removed. The on-column detection window was burned with a thermal stripper (Unimicro Technologies, Pleasanton, CA, USA) under a pressure of 6000 psi. A typical SEM image of a cross-section of a packed 100 μm i.d. capillary prepared using the experimental method described above is shown in Figure 2A. The UV/Vis detection window and the inlet frit tip of the column are shown in Figure 2B,C.

## 4. Conclusions

In this work, we systematically investigated the separation performances of columns packed with 300, 420, 500, 620, and 800 nm C18-SiO_2_ particles for aromatic compounds, PAHs, estrogens, and proteins (lysozyme, cytochrome C, ribonuclease A, and ovarian cancer anti-idiotypic mini body) on a pCEC-UV/Vis platform. EOF played a major role in driving the mobile phase in the columns packed with submicron particles, providing a high-speed and extremely efficient separation of the seven aromatic compounds, eight PAHs, five estrogens, three proteins, and anti-idiotypic mini body. Good repeatability and high separation efficiency were obtained using our packed column. However, more studies are needed to improve the efficiency of the column preparation, which is also the most challenging part of realizing the wide application of this column packed with submicron particles. In conclusion, our study demonstrated that the separation system in which a column was packed with submicron silica particles, coupled with pCEC, has great promise for protein and complex biological sample analyses.

## Data Availability

The data presented in this study are available from the corresponding author upon request.

## Supplementary Materials

The following supporting information can be downloaded at: https://www.mdpi.com/article/10.3390/molecules28083542/s1, Figure S1: Column efficiency curves of thiourea and six aromatic compounds in 300, 420, 500, 620, 820 nm non-porous submicron packed columns. Peaks: (1) Thiourea; (2) a-naphthol; (3) benzophenone; (4) naphthalene; (5) biphenyl; (6) butylbenzene and (7) phenylcyclohexane; Figure S2: pCEC separation of eight PAHs at voltages of 2 kV to 16 kV on the columns packed with 300, 420, 500, 620, and 820 nm particles. Experimental conditions: Column: 100 μm i.d. × 100/300 mm (effective/total length); Pressure = 15.5 MPa; Mobile phase: acetonitrile–10 mM phosphate butter (60:40, *v*/*v*); pH = 7.8; Split ratios 800:1. Peaks: (1) Naphthalene, (2) Acenaphthylene; (3) Fluorene; (4) Phenanthrene; (5) Anthracene; (6) Fluoranthene; (7) Benzanthracene and (8) Benzofluoranthene; Figure S3: Column efficiency curves of eight PAHs on the columns packed with 300, 420, 500, 620, and 820 nm particles. Peaks: 1. naphthalene; 2. acenaphthylene; 3. fluorene; 4. luxuriant; 5. anthracene; 6. fluoranthene; 7. benzanthracene; 8. Benzofluoranthrene; Figure S4: Column efficiency curves of five Estrogens in 300 nm non-porous submicron packed column. (1) E3; (2) BPA; (3) E2; (4) E1; (5) HE; Table S1: The repeatability (three replicates, columns packed by 300, 420, 500, 620, 820 nm nonporous particles) of naphthalene and seven PAHs in pCEC (n = 3).

## Sample Availability

Samples of the compounds are not available from the authors.
