# Supplementary material for: Submicron Nonporous Silica Particles for Enhanced Separation Performance in pCEC
Source: Molecules. 2023 Apr 17;28(8):3542. doi: 10.3390/molecules28083542 (PMC10145033; doi:10.3390/molecules28083542)
Supplement: Supplementary file 1 [file molecules-28-03542-s001.zip › molecules-2245983-supplementary.pdf]

Supplementary Material for

Submicron nonporous silica particles for enhanced separation performance in pCEC

Qing Liu[1,2], Chao Yan[2]* and Yan Wang[2]*

[1] Hubei Province Key Laboratory of Occupational Hazard Identification and Control, School of Medicine, Wuhan University of Science and Technology, Wuhan 430065, China

[2] School of Pharmacy, Shanghai Jiao Tong University, Shanghai 200240, China

Corresponding Author

* Phone: +86-021-3420-5988.Fax: +86-021-3420-5908
E-mail: chaoyan@sjtu.edu.cn (Chao Yan).

* Phone: +86-021-3420-5673.Fax: +86-021-3420-5908
E-mail: wangyan11@sjtu.edu.cn (Yan Wang).

Table S1. The repeatability (three replicates, columns packed by 300, 420, 500, 620, 820 nm nonporous particles) of naphthalene and seven PAHs in pCEC (*n=3*).

PAHs	300 nm				420 nm				500 nm				620 nm				820 nm			
	t_R[a] (min)	RSD (%)	A[b](μV·s)	RSD (%)	t_R (min)	RSD (%)	A (μV·s)	RSD (%)	t_R (min)	RSD (%)	A (μV·s)	RSD (%)	t_R (min)	RSD (%)	Avrea (mV/s)	RSD (%)	t_R (min)	RSD (%)	A (μV·s)	RSD (%)
Naphthalene	2.87	1.20	1081	0.32	2.94	0.62	2337	1.21	2.66	0.02	950	0.63	3.50	0.26	5228	0.07	4.32	0.25	1047	4.63
Acenaphthylene	3.04	1.61	2164	1.18	3.19	0.61	3063	1.25	2.78	0.02	1287	0.89	3.79	0.22	5123	1.19	4.65	0.30	1467	4.91
Fluorene	3.45	0.62	2464	0.97	3.59	0.60	3761	1.82	3.06	0.02	1592	1.01	4.31	0.22	6229	0.95	5.33	0.41	1747	6.09
Phenanthrene	3.69	0.16	3986	1.46	3.95	0.59	4797	0.97	3.24	0.04	1949	2.05	4.72	0.17	7814	1.14	5.83	0.50	2519	2.04
Anthracene	3.87	0.62	3251	0.02	4.18	0.59	3103	1.60	3.42	0.02	1182	3.64	4.99	0.15	6975	1.27	6.22	0.52	2135	0.89
Fluoranthene	4.34	0.20	2967	0.51	4.81	0.56	2990	2.29	3.73	0.05	1145	3.14	5.74	0.12	5034	1.88	7.13	0.66	1418	2.50
Benzanthracene	5.76	0.65	2980	3.17	6.80	0.61	2911	0.70	4.93	0.04	667	4.05	7.97	0.21	4133	4.91	10.25	0.95	1241	4.63
Benzofluoranthene	7.58	0.04	1525	2.26	9.39	0.75	3536	2.04	6.45	0.04	449	0.56	10.76	0.40	4182	7.40	14.23	1.30	1457	1.13

[a] tR (min) = Average Retention time.

[b] A (mV/s) = Average Peak area.

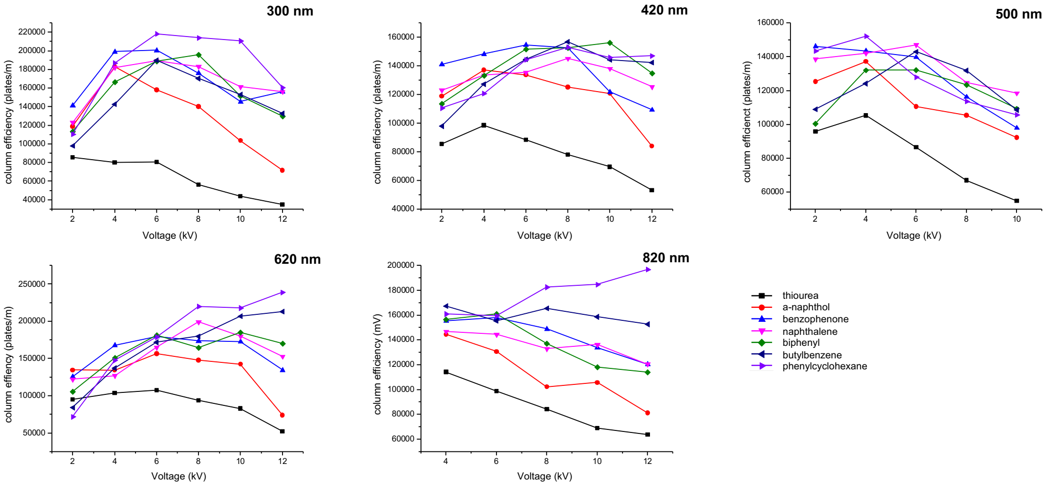

Figure S1. Column efficiency curves of thiourea and six aromatic compounds in 300, 420, 500, 620, 820 nm non-porous submicron packed columns. Peaks: 1) Thiourea; 2) a-naphthol; 3) benzophenone; 4) naphthalene; 5) biphenyl; 6) butylbenzene and 7) phenylcyclohexane.

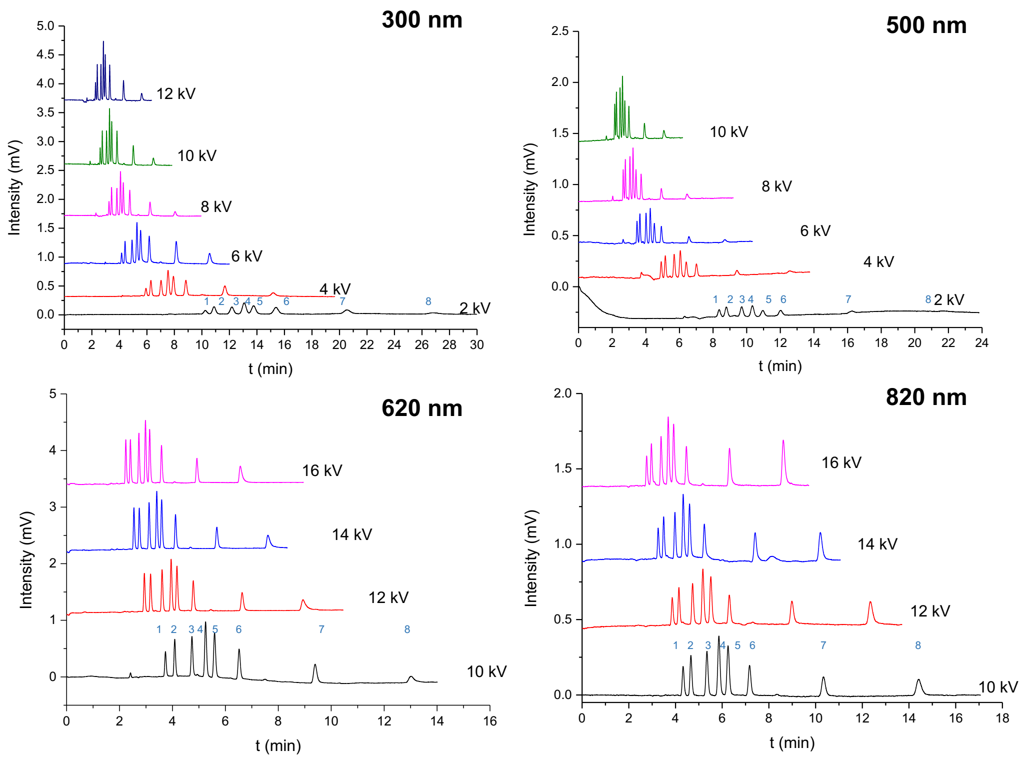

Figure S2. pCEC separation of eight PAHs at voltages of 2 kV to 16 kV on the columns packed with 300, 420, 500, 620, and 820 nm particles. Experimental conditions: Column: 100 µm i.d. x 100/300 mm (effective/total length); Pressure = 15.5 MPa; Mobile phase: acetonitrile–10 mM phosphate butter (60:40, v/v); pH = 7.8; Split ratios 800:1. Peaks: 1) Naphthalene, 2) Acenaphthylene; 3) Fluorene; 4) Phenanthrene; 5) Anthracene; 6) Fluoranthene; 7) Benzanthracene and 8) Benzofluoranthene.

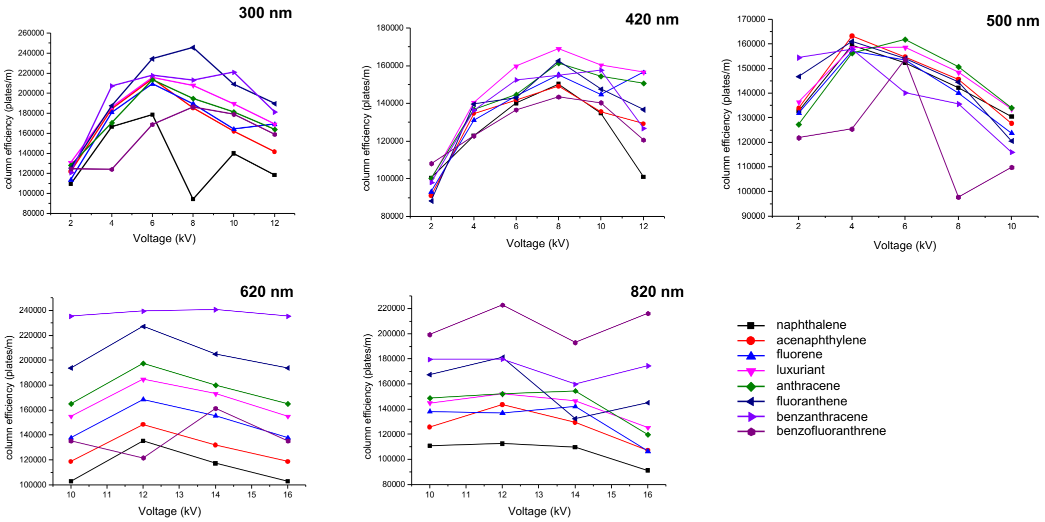

Figure S3. Column efficiency curves of eight PAHs on the columns packed with 300, 420, 500, 620, and 820 nm particles. Peaks: 1. naphthalene; 2. acenaphthylene; 3. fluorene; 4. luxuriant; 5. anthracene; 6. fluoranthene; 7. benzanthracene; 8. Benzofluoranthrene.

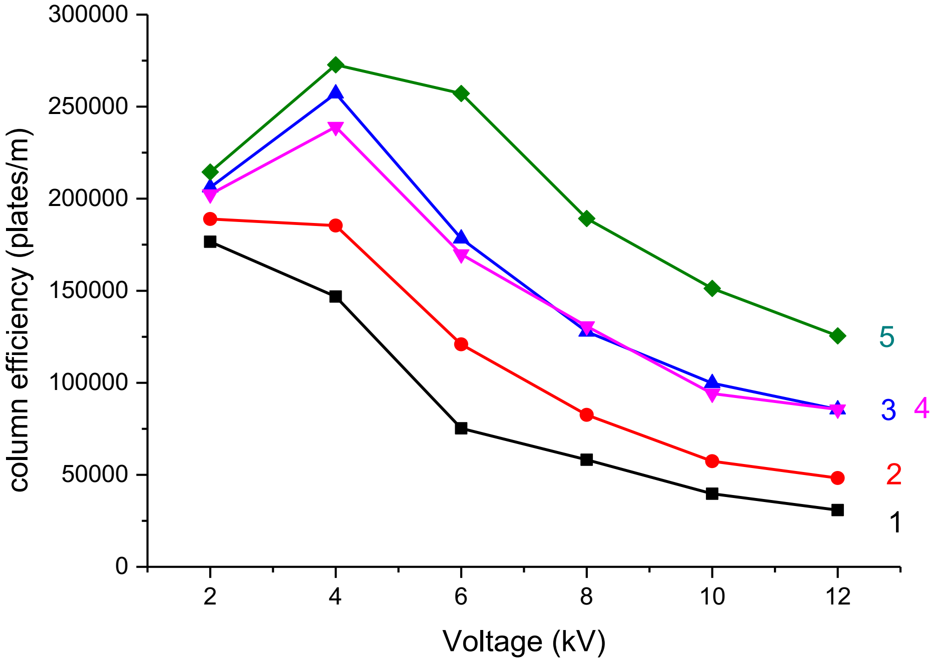

Figure S4. Column efficiency curves of five Estrogens in 300 nm non-porous submicron packed column. 1) E3; 2) BPA; 3) E2; 4) E1; 5) HE.
